# Acoustic Identity: Linking Signature Whistles and Visual Identification in a Threatened Dolphin Population

**DOI:** 10.3390/ani15223259

**Published:** 2025-11-10

**Authors:** Amber Crittenden, Kate Robb, Christine Erbe

**Affiliations:** 1 Marine Mammal Foundation (MMF), Hampton East, Melbourne, VIC 3195, Australia; kate@marinemammal.org.au; 2 Centre for Marine Science and Technology, Curtin University, Bentley, Perth, WA 6102, Australia; c.erbe@curtin.edu.au

**Keywords:** signature whistle, dolphin, Burrunan, *Tursiops australis*, Gippsland Lakes

## Abstract

Signature whistles (SW) are a significant element of the vocal repertoire of delphinid species. They encode self-identifying information and allow social cohesion between related and allied individuals through often complex, stereotyped whistle contours. SW recordings allow researchers to explore the presence of individual animals, understand social dynamics, and estimate population size without visual observation. This represents the first study aiming to match distinct SW contours to individual Burrunan dolphins (*Tursiops australis*) in the Gippsland Lakes, Victoria, Australia. Simultaneous photographic identification and in situ hand-held hydrophone recordings collected in 2021–2024 contained 57 individual dolphins and 236 whistles, resulting in 22 unique SW contours being extracted following modified SIGnature IDentification (SIGID) criteria. Conditional probability analyses revealed that most SW contours were associated with clusters or pairs of dolphins, rather than individuals, reflecting the species’ fission–fusion social structure. Cluster analysis supported these associations, highlighting the difficulty in isolating SWs in a population where individuals are not observed in isolation. Additionally, social structure, philopatric behaviour, and the acoustic environment of transient individuals are suggested to influence signature whistle production and complexity. Although direct signature whistle-to-individual matching remains limited, the study demonstrates that population-level and subgroup-level monitoring by passive acoustic methods is achievable. This approach provides a critical acoustic tool for conservation management of the small, threatened Gippsland Lakes Burrunan dolphin population and offers a foundation for future study of individuals through passive acoustic monitoring.

## Simple Summary

Acoustic identity is an essential element of dolphin interaction, achieved through the production of a “signature whistle” unique to each individual. These whistles are often complex and emitted repetitively. Using distinct whistle characteristics, researchers have identified 22 unique signature whistles within acoustic recordings collected in the presence of 57 Burrunan dolphins. The identified signature whistles were found to be associated with clusters or pairs of Burrunan dolphins, as the highly social nature of dolphin population structures prevents individuals from being recorded separately. Further, female dolphins and dolphins exhibiting transient behaviours were found to have very similar signature whistles. Identification of these signature whistles in acoustic datasets recorded passively will allow for improved population monitoring and ultimately conservation management.

## 1. Introduction

### 1.1. Signature Whistles

Delphinids produce a variety of sounds for key behavioural purposes, including navigation, prey capture, and communication [1]. One of these is the whistle sound type: a frequency-modulated tonal signal used in social, group-cohesion contexts [2]. It is believed that significant information is encoded in the modulation of the whistle contour, with numbers of steps, inflection points, extrema, and overall contour shape linked to different behaviours [3,4]. The signature whistle (SW) is believed to be the most common whistle produced by bottlenose dolphins [5] and is associated with self-identification [6], typically distinguishable from non-SW emissions by its stereotypy and additional non-linear phenomena [7].

Bottlenose dolphin calves learn their SW from their mothers [8], with contours remaining highly stable over time once developed to preserve individual identification [9]. Mothers are documented to produce their SW almost constantly in the presence of their calf to maintain and strengthen the mother–calf bond [10,11]. Female calves develop and differentiate their SW significantly from their mother’s, whereas male SWs remain similar [12]. This is believed to be due to the bottlenose dolphin population’s social structure, wherein females remain with large nursery pods of multiple generations for optimised resource distribution and knowledge sharing from matriarchs [13,14]. Differentiation between mother and daughter SWs is thus required for acoustic identification within the pod. Contrastingly, males typically establish new alliances [15,16]. Therefore, SW presence within acoustic datasets may elucidate pod structure and social dynamics, with potential for identifying individuals and estimating group size using sound alone.

The SIGnature IDentification (SIGID) method outlined in Janik et al. [17] identifies several criteria for assessing whether a recorded whistle is a SW. SIGID has since been employed and modified by researchers across the globe, with consideration given to how population dynamics, soundscape contributors, and geographical composition might affect SW structure [18,19,20]. Namely, the number of repetitions of the same whistle contour (a SW ‘bout’), the duration between individual whistles (the inter-whistle interval, IWI), and the degree of modulation or ‘uniqueness’ of the whistle shape are included as indicators for SIGID within acoustic datasets. The specifics of these criteria are often unique to the species, location, and population being studied [19,21,22].

### 1.2. Burrunan Dolphin

The Burrunan dolphin (*Tursiops australis*) is a recently described species found in southern and south-eastern Australia [23,24,25,26,27]. One of the two known Victorian resident populations is located in the Gippsland Lakes (GL) [26]. This population is small, genetically isolated, and exposed to various anthropogenic pressures, including physical disruption (i.e., vessel approaches, disturbances, and strikes) [28], environmental degradation (i.e., anthropogenic contaminants and habitat loss) [29,30,31,32,33], disease [34], and anthropophonic noise (anthropophony). While little is yet known about the behavioural ecology of this species, other small, coastal delphinid populations similar to the Burrunan dolphin exhibit highly complex social structures. These structures include several levels of association between individuals of the same sex, such as males forming cooperative pairs (‘dyads’) or trios (‘tryads’), while females form ‘nursery pods’ of mothers with calves of similar ages [35,36,37,38].

Characterisation of the acoustic repertoire of the Burrunan dolphin has recently been undertaken [39]. The species has been found to emit six general classes of whistles and four classes of burst-pulse sounds [39]. Whistle repertoire composition of the Burrunan dolphin resembles those of similarly structured delphinid populations (coastal, urbanised environment, small population number) around Australia, though it showed variation in the overall proportion of whistle class use, indicating some species-level differentiation [39]. Additionally, the GL population exhibited a significantly more diverse repertoire of vocalisations (greater frequency range and larger standard deviation of measured characteristics) than that of their Port Phillip Bay counterparts, suggesting acoustic pressures or influences within the GL and surrounding environment were absent in the other Victorian population [39].

The GL population of Burrunan dolphins includes several transient animals, believed to migrate seasonally through Bass Strait [26], resulting in periodic fluctuations in GL population size, particularly in the winter. However, the GL population has experienced several mortality events linked to influxes of freshwater into the estuarine Lakes system [34], with the most recent freshwater-related event occurring during the prolonged La Niña period of 2020–2023 [40]. Importantly, the Burrunan dolphin exists as an ecosystem sentinel within the complex biodiversity of the ecologically significant GL, a Ramsar wetland-listed habitat [41]. Therefore, the GL Burrunan dolphin population has been targeted for conservation management strategies, with prioritisation of population abundance estimates and identification of extant individuals.

### 1.3. Rationale and Study Aim

Population assessment is essential for monitoring species abundance or decline. While this is typically conducted via visual surveys, traditional boat- or land-based study is restricted to daylight hours and fair-weather conditions. Given baseline species vocalisation assessment has been conducted [39]; identifying not only species presence but the presence of specific individuals through non-invasive methods such as passive acoustic monitoring (PAM) enables researchers to monitor populations and individual animals remotely. However, this is only possible once sufficient cataloguing of individuals and their SWs has been completed. Then, SWs within acoustic datasets may be used to estimate population abundance and distribution [18,22], track individual welfare [42], and monitor ecological impact [43].

This study seeks to identify SW contours, following modified SIGID criteria, within the GL Burrunan dolphin population. SWs will be matched to GL Burrunan dolphins using photographic fin identification (mark-recapture) data. Future identification and monitoring of the population at the individual level via PAM in the Gippsland Lakes will then be possible, broadening response to conservation and species persistence concerns.

## 2. Materials and Methods

### 2.1. Fieldwork

#### 2.1.1. Study Site

**Figure 1:**
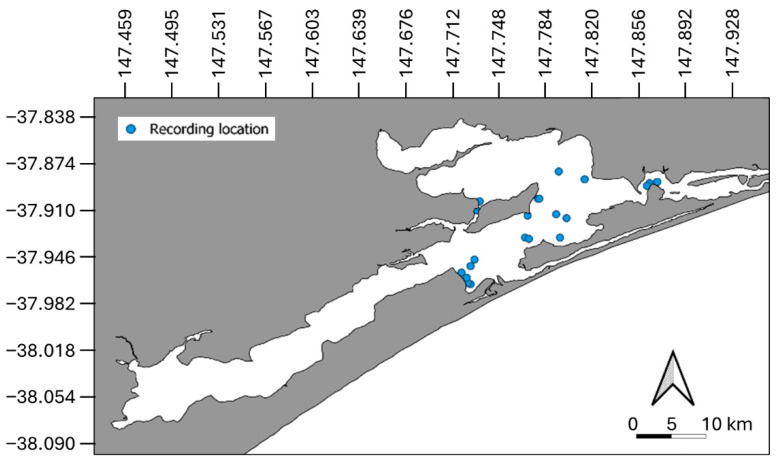
Map of the Gippsland Lakes with locations of acoustic recording indicated by blue dots.

GL is located on the coast of south-eastern Victoria, Australia, ~270 km from the state’s capital, Melbourne (Figure 1). It is a series of interconnected lakes, lagoons, and marshes comprising ~600 km^2^ of waterway [44], with depths varying from 2 m to 14 m across the three main lakes (average depth of 4 m). A small (80 m) artificially maintained entrance to Bass Strait allows for consistent saltwater input into the Lakes at its most north-eastern point, with periodic saltwater inputs from natural openings in the southern bank experienced during storm water surges and king tides [31,41,44]. Five main rivers input freshwater from rain and snowmelt into the lakes year-round [45]. The system is subject to seasonal vessel traffic, including recreational powered (boats, jet skis) and unpowered (yachts, stand-up paddle boards, kayaks) vessels, and several small passenger ferries/tour operators, and hosts a thriving fishing industry. It is surrounded by agricultural farmland, industrial zoning, and national parkland. Given the dynamic nature of the system, a diverse ecosystem of oceanic and freshwater species of plants, fish, birds, and mammals has evolved in GL.

#### 2.1.2. Data Collection

Seasonal boat-based surveys of the GL Burrunan dolphin population (residents and transients) were undertaken during daylight hours in calm water (<15 kts) to ensure visibility, in 2021–2024. Surveys were conducted from a 2C research vessel, a 5.7 m Ensign 570 powered by a 90-hp Mercury outboard engine. Established survey transects were followed throughout the system until a pod of dolphins was encountered (a ‘sighting’), where the transect was paused and the dolphins approached [27].

A ‘focal follow’ was conducted wherein start and end time were recorded, photographic images of the dolphin dorsal fins (‘fin ID’) of the GL Burrunan dolphins were collected (in line with a long-term mark-recapture study), ensuring both left-hand-side (LHS) and right-hand-side (RHS) images of the dorsal fin were captured, and periodic updates were dictated. Fin ID images were captured using two digital SLR cameras: a Canon EOS 6D (Canon Inc., Tokyo, Japan) with a Tamron Di VC USF 100–400 mm f/4.5–6.3 zoom lens, and a Nikon D750 (Nikon Corporation, Tokyo, Japan) with a Tamron Di II VC HLD 18–400 mm f/3.5–6.3 zoom lens. Periodic updates included GPS location (wherein boat location equated to dolphin location, thus recording the ‘track’ of the dolphins), predominant and secondary behaviour (travel, mill, feed, or social behaviour), pod composition (number of subgroups, degree of association, total pod spread [m]), water depth [m], and water temperature [°C], recorded every 5 min on a Dictaphone for later transcription [28]. Further 30 min updates on the presence of other vessels, visibility (poor–excellent), glare (percentage of horizon interrupted), sea state (Beaufort), and cloud cover (Octa), were also recorded. Long-term population monitoring determined sex and population demographic of individuals through biopsy sampling [26] and a mark-recapture study, i.e., mother–calf associations [40]. Sightings and their associated focal follows were concluded at the onset of the first of three possible events: (a) 2 h focal-follow duration was achieved; (b) dolphins exhibited avoidance behaviours, or (c) a change in environmental conditions resulted in suspension of survey. In the case of a or b, the survey transect was recommenced from the end location of the sighting, such that discovery of another dolphin pod would be possible and a representative sampling of the population was achieved.

Where possible, acoustic vocalisations were recorded with a handheld CR-80-40 hydrophone (Burns Hydrophone Systems, Nelson Bay, NSW, Australia; sensitivity: –166 ± 5 dB re 1 V/μPa; frequency range: 7 Hz–80 kHz) deployed over the side of the vessel, with sound files recorded on a H5 Zoom Handy Recorder (Zoom Corporation, Tokyo, Japan), sampling frequency 96 kHz [39]. Recordings were taken with the vessel positioned as close as possible to the observed pod (defined as any two or more dolphins associating or interacting within 100 m of one another [46]) without causing disturbance, with the vessel engine switched off. Recordings were listened to in real-time to ensure effective vocalisation capture. Recording ceased and the vessel was repositioned if current/wind or behaviour resulted in a distance > 200 m from the dolphins (Figure S1).

### 2.2. Data Preparation

#### 2.2.1. Photo-Identification

**Figure 2:**
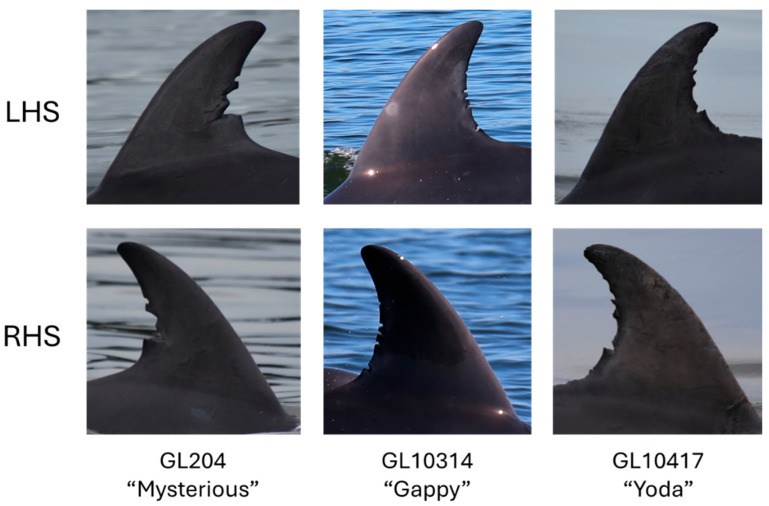
Examples of high-quality cropped and sorted left-hand-side (LHS) and right-hand-side (RHS) fin ID images for three animals in the Gippsland Lakes Burrunan dolphin population.

Individual dolphin identification, within each sighting, was made by observing the unique series of permanent nicks and notches on the trailing edge of the dolphin’s dorsal fin [47,48]. Photographs were processed according to the Marine Mammal Foundation’s (MMF) Fin Identification Protocol, as outlined in [40], which was developed based on methodology by Urian et al. [48]. In summary, all raw photographs were assessed based on focus, contrast, angle of dorsal fin, and proportion of fin captured [48,49], with the highest-quality fin ID images of the identifiable animals within each sighting retained, and the removal of poor-quality images (blurry photos, water shots, images with overlapping animals) in a culling process. Photos were cropped such that each dorsal fin occupied its own image, allowing individuals to be ‘captured’ in their own image. Cropped fin ID images were sorted into folders of individuals based on identifying nicks and notches on the dorsal fin and grouped by sighting. Dolphin identifications were later verified by an expert observer against a long-term catalogue of individuals, coded based on the locations of fin markings. At least one LHS and one RHS image of each marked dorsal fin was retained (Figure 2). A spreadsheet was compiled detailing each image file name, date, time, sighting number, and ID code for the individual photographed.

#### 2.2.2. SW Identification and Extraction

**Figure 3:**
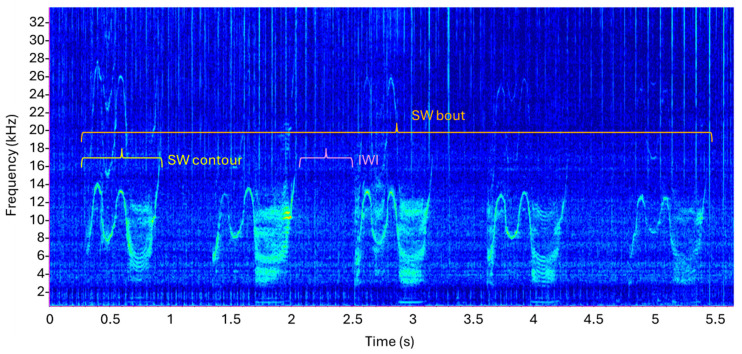
Example spectrogram of a SW bout (orange) i.e., five repetitions of the SW contour (yellow) with 50% of repetition IWI (pink) 0.5–10 s.

The SWs were identified visually and aurally within hydrophone recordings using modified SIGID criteria [17], as employment of traditionally conservative SIGID criteria excluded several SW contours of sufficient quality (i.e., strong signal-to-noise ratio, clear start and end points) from analysis. Bouts of SWs were defined as repetitions of three or more of the same distinct contour (i.e., stereotyped whistles of the same whistle class that possessed the same or very similar characteristics, including bandwidth, duration, and modulations such as numbers of extrema, inflection points, and steps; see Erbe and Wei [50] for definitions), where IWI for at least 50% of the repetitions was 0.5–10 s (Figure 3). Whistles of the same contour that did not meet the SW bout criteria were not considered SWs and therefore excluded from analysis.

Once bouts had been identified, the characteristics of the contour (start and end frequency, minimum and maximum frequency, start and end time, and numbers of extrema, inflection points, and steps) of each whistle within the bout were extracted in the selection table function of Raven Pro (version 1.6.5; Cornell Laboratory of Ornithology, Ithaca, NY, USA). Spectrograms were created in windows of 512 and 1024 samples, Hamming windowed, with 50% overlap. Images of each bout were extracted and compared, with a label for each type of SW contour assigned based on the measured spectral features as well as overall contour shape and presence of burst-pulse sections or biphonics. Selection tables of extracted whistles were compiled into a single spreadsheet with their corresponding SW contour label, the date and time of recording, and the sighting during which it was recorded, following methods outlined in Erbe et al. [51].

### 2.3. Data Analysis

Spreadsheets of dolphin fin ID image and SW contour measurements were compiled into a single master document, grouping individuals sighted and the SW contours recorded by sighting. Statistical analyses were conducted to determine whether SW contours could be attributed to individual animals.

All statistical analysis was conducted in MATLAB (version R2021b; The MathsWorks Inc., Natick, MA, USA) following methods outlined in Erbe et al. [51]. SW bouts were considered only as a criterion for the identification of SWs. Each of the whistles within an SW bout was considered individually in statistical analysis, such that the exclusion of whistles of insufficient quality (i.e., weak signal-to-noise ratio, indeterminable start and end points) did not confound results. Descriptive statistics, including means, standard deviations, ranges, and percentiles, of the measured whistle characteristics (start frequency [kHz], end frequency [kHz], minimum (min) frequency [kHz], maximum (max) frequency [kHz], duration [s], and numbers of extrema, inflection points, and steps) were then computed. A dendrogram of association between animals was produced to provide insight into the social bonds of the GL Burrunan dolphin population. Of note, this is not a comprehensive analysis of social alliances within the GL population, as only sightings wherein SWs were recorded were considered. As such, traditional requirements and reporting metrics associated with the study of social alliances have been excluded here. Conditional probabilities of observing individual animals while recording each of the SW contours, as well as those of recording any of the SW contours in the visual presence of each of the dolphins, were generated and presented as histograms. Lastly, given high levels of association between animals and the consequent grouping of SW contours, an agglomerative hierarchical binary cluster tree (distance: Euclidean, linkage: average) was computed to determine whether relationships existed between SW contours and groups of individuals. A post hoc cophenetic correlation coefficient was computed for both animal linkage and whistle linkage, determining the reliability of pairwise distance preservation during analysis.

## 3. Results

A total of 22 acoustic recordings of Burrunan dolphin vocalisations collected between 2021 and 2024 throughout GL were explored in this study, comprising 2 h 20 min of recording time. Recordings were collected across 12 different days encompassing 13 individual dolphin sightings, culminating in 25 h 29 min of survey effort (Table S1). Within these sightings, 6168 images were taken, resulting in 57 dolphins identified (Table S2).

### 3.1. Sighting Associations

**Figure 4:**
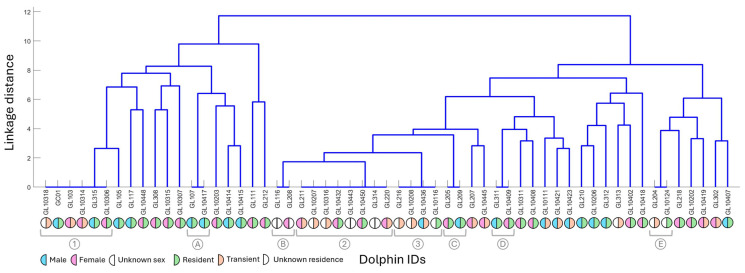
Dendrogram of dolphin association by identification within sightings, with linkage distance on the *y*-axis (lower values indicate closer linkage; leaves along the *x*-axis indicate clusters and pairs), and animals observed on the *x*-axis. Demographic information for animals observed includes sex (male = blue, female = pink, unknown = blank) and residency (transient = orange, resident = green, unknown = blank). Social clusters identified are denoted by numbers 1–3, and social pairs are denoted by letters A–E.

No animal was ever observed alone, and groups of the same animals were repeatedly observed when SW were recorded. The level of association between individuals in the 13 sightings is demonstrated in Figure 4. Three clusters (denoted by numbers 1–3) and five pairs (denoted by letters A–E) of dolphins consistently seen together when SW were recorded were identified. Cluster 1 comprised GL315, GC01, GL103, GL10318, GL10314, and GL10306 (two resident males, one resident female, two transient females, and a transient animal of unknown sex). Cluster 2 comprised GL211, GL10443, GL10450, GL10432, GL220, GL10316, GL314, and GL10207 (two transient females, two resident females, two transients of unknown sex, and two animals of unknown sex or residence). Cluster 3 comprised GL216, GL10208, GL10436, and GL10116 (one transient male, one resident of unknown sex, and two transients of unknown sex). Pair A consisted of GL107 and GL10417 (established dyad of two resident males). Pair B consisted of GL116 and GL208 (both of unknown residence, one of unknown sex, one female). Pair C consisted of GL205 and GL209 (a female and a male resident, both deceased). Pair D consisted of GL311 and GL10409 (male calf and older female, both residents). Finally, Pair E consisted of GL204 and GL10124 (a transient and a resident, both of unknown sex).

### 3.2. Signature Whistle Contours

**Figure 5:**
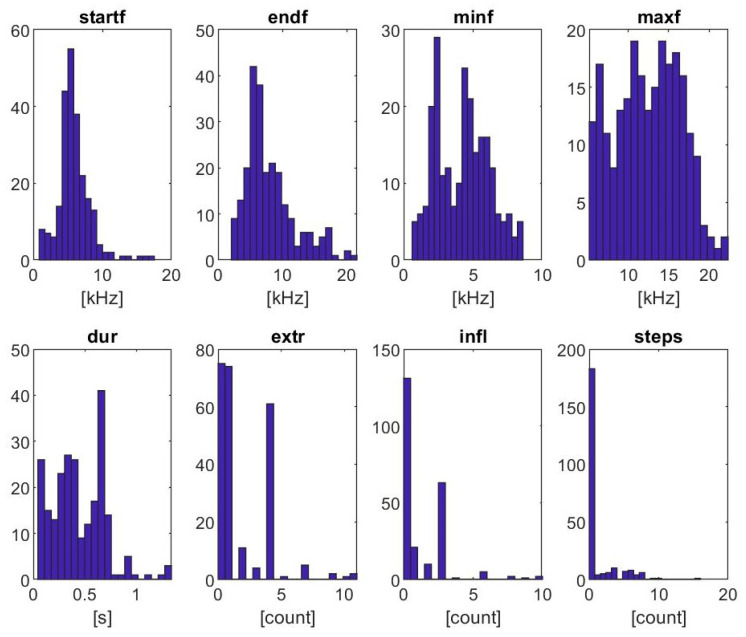
Histograms of the measured parameters of the whistles within SW bouts recorded in the Gippsland Lakes Burrunan dolphins 2021–2024; start frequency (startf), end frequency (endf), minimum frequency (minf), maximum frequency (maxf), duration (dur), extrema (extr), number of inflection points (infl), and number of steps (steps).

Acoustic recordings contained 54 identifiable SW bouts complying with the adjusted SIGID criteria. Within the bouts, 236 individual whistles were extracted. Descriptive statistics for these whistles are presented in Table 1. Whistles within SW bouts ranged from 0.61 to 22.34 kHz, had a mean bandwidth of 4.24–12.43 kHz, and often were without inflection points or steps. Large standard deviations in SW modulations (numbers of extrema, inflection points, and steps) indicate the complexity included in SW contour production. Further, histograms of the measured parameters demonstrate that frequency measurements were Gaussian distributed, while numbers of extrema, inflections, and steps were approximately Poisson distributed (Figure 5).

From the SW bouts, 22 unique SW whistle contours were identified for correlation analysis (Table S3). SWs comprised three of the six previously defined whistle classes produced by the Burrunan dolphin [39]: upsweep, convex, and sine class whistles.

### 3.3. Conditional Probability

Little insight was provided when considering how the presence of individual dolphins within a sighting affected the probability of a particular SW contour being recorded (Figure S2). The ShortConvex contour was mostly associated with the presence of GL218, though no other SW contour indicated a direct correlation with a particular dolphin. The HookUp and WUp contours indicated a correlation with only two dolphins each: both GL111 and GL10307, and GL212, respectively. The MBuzz and the LongUp contours demonstrated no direct correlation to any one dolphin, reflecting their high proportional presence within sightings (26.27% and 7.20%, respectively). The LowUp and ZUp contours both had high affinity for the same four dolphins: GL111, GL308, GL10302, and GL10315. Many of the SW contours were consistently recorded in the presence of seven or more dolphins, including the BConvex, BuzzConvex, FlatConvex, InflConvex, SmoothConvex, StepConvex, HookSine, MultiL, StepSine, BuzzUp, ConstUp, InflUp, MidUp, and UpFlat contours, preventing their pairing with individual dolphins.

**Figure 6:**
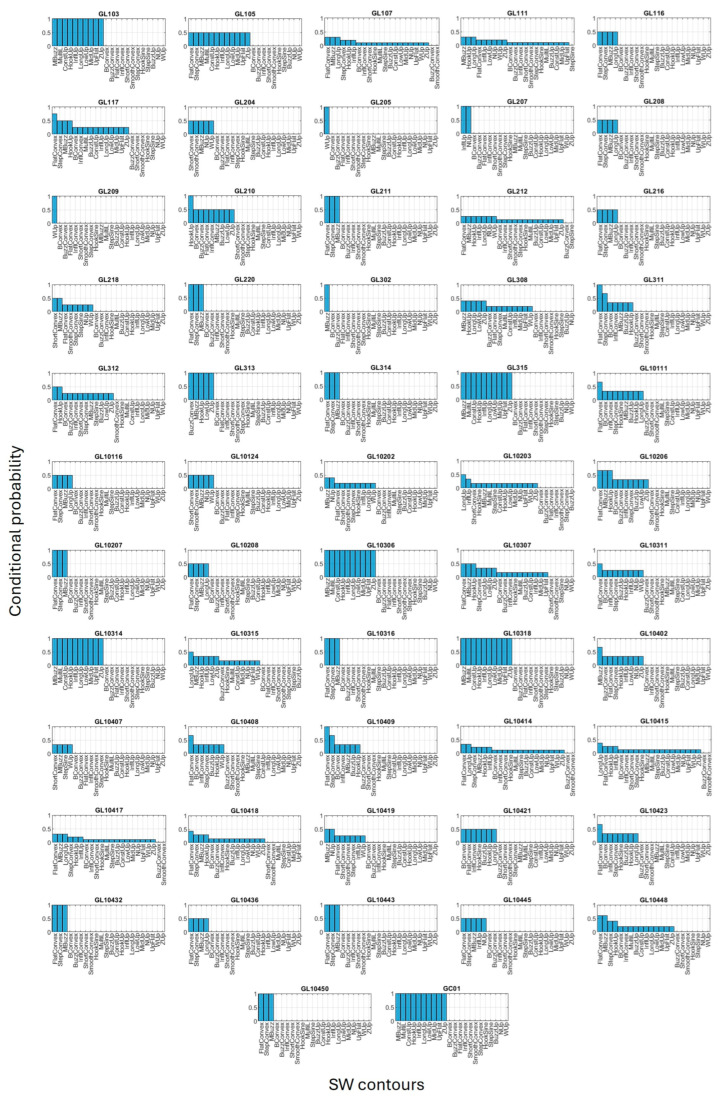
Histograms of the conditional probability of the SW contours being recorded in the presence of each dolphin.

When examining how the recording of a particular SW contour might indicate the likelihood of a dolphin being present within a sighting, some cohesion between individuals and contours was seen (Figure 6). Five dolphins displayed a direct relationship between their presence and the probability of a single SW contour being recorded: GL205, GL209, GL210, GL302, and GL10409. While some dolphins displayed conditional probability that linked them to two or three possible SWs, six dolphins were consistently present when 10 specific SW contours were emitted: GL103, GL10306, GL10314, GL10318, GL315, and GC01, indicating they were likely vocalising during this time, but preventing definite matching of contours to these animals. This can be attributed to their high level of cluster and pair association within sightings (Section 3.1). Nine of the observed dolphins were unlikely to have had their SW recorded in this dataset, as indicated by the low (<0.5) conditional probability of any of the SW contours being emitted in their presence: GL107, GL111, GL308, GL10202, GL10407, GL10414, GL10415, GL10417, and GL10418.

### 3.4. Clustering Contours and Individuals

**Figure 7:**
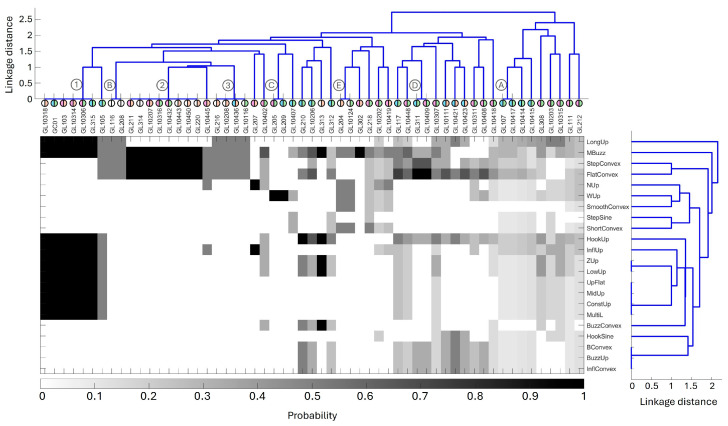
Dendrograms of the frequency of simultaneous occurrence of dolphins (top) and SW contours (right), with the matrix of posterior probabilities of recording each SW contour in the presence of each dolphin aligned between. Clusters and Pairs from Section 3.1 identified in grey circles. Population demographics from Section 3.1 identified in coloured circles.

Given that conditional probabilities did not reveal distinct SW contour-dolphin associations, cluster analysis involving dendrograms of both dolphin sighting (cophenetic correlation coefficient: 0.892) and SW contour (cophenetic correlation coefficient: 0.895) recording, aligned by probability of occurrence, instead highlighted that groups of individuals could be associated with groups of SW contours (Figure 7). Of those identified in Section 3.1, the sighting of two clusters and two pairs (Figure 7 top) were revealed to be linked to the recording of the same contours (Figure 7 middle). The presence of Cluster 1 was most associated with the LongUp, MBuzz, HookUp, InfluUp, ZUp, LowUp, UpFlat, MidUp, ConstUp, and MultiL SW contours. These associations are reflected in the SW contour dendrogram (Figure 7 right), with the ZUp and LowUp contours, as well as the UpFlat, MidUp, ConstUp, and MultiL contours showing high association. Cluster 2 was also highly associated with the MBuzz contour, as well as the StepConvex and FlatConvex contours. Both Cluster 1 and Cluster 2 represent primarily transient female individuals. Pair D was also associated with the FlatConvex contour, while Pair C was the only group associated with the WUp contour. These two pairs comprise only resident individuals; again, the majority are females.

Other individuals that showed extremely high conditional probability overlap between observability and SW contour recording included GL207 (transient female) with the NUp and InflUp contours; GL210 (resident male) with the HookUp contour; GL313 (transient individual) with the MBuzz, HookUp, ZUp, and LowUp contours; and GL302 (transient female) with the MBuzz contour.

## 4. Discussion

This study involved the simultaneous recording of Burrunan dolphin SWs and photographic fin ID images in the Gippsland Lakes (GL), Victoria, Australia, aiming to align unique SW contours with individual dolphins. In the 2 h 20 min of acoustic recordings collected in 2021–2024, the 57 dolphins photographed and identified in the 13 dolphin sightings were never observed in isolation, resulting in the 22 distinct SW contours being associated with several possible individuals. Observed social groups (i.e., social clusters and pairs), rather than individuals, were matched with SW contours.

### 4.1. Fission–Fusion

The clusters and pairs of dolphins regularly observed together, and the high degree of association within them, suggest that GL Burrunan dolphins are highly selective about the individuals with which they associate. While the formal social alliances and structure of the entire population over the long-term study are currently undergoing assessment [40], we provide here the first evidence of social groupings that are highly associated when SW are recorded, that is, where the two datasets (acoustic and fin ID) overlap. The highly social nature of bottlenose dolphins is believed to be a key driver in vocal plasticity and vocal structure formation [52]. Findings herein indicate that the population demonstrates a ‘fission–fusion’ dynamic between subgroups, but rarely between individuals, as outlined in the observed social clusters and pairs. Consequently, the same SW contours will therefore always be recorded when these groups of highly associated individuals are observed [20], preventing alignment of the SW contour and the individual dolphin. Cook et al. [53] found that SWs were the most commonly emitted whistle in isolated dolphins, achieving specific dolphin and SW contour association. However, repeated whistle emission in isolated bottlenose dolphins may be associated with stress [54]. A collection of acoustic recordings of solitary animals or mother–calf pairs that are opportunistically observed during seasonal surveys should be prioritised.

### 4.2. Male–Female

Dolphin social structure involves subgroup formation based on sex, with female offspring remaining with their mothers and other female relatives into adulthood [55]. Consequently, female calves must quickly develop unique SW contours to differentiate themselves from their female relatives [56]. Yet, despite the formation of district SW contours, these strong matrilineal social alliances prevent isolated recording of individual female SW contours and identification of individuals responsible for these SWs. The strong social grouping of females during sightings of recorded SWs in this dataset, particularly in Clusters 1 and 2, prevented alignment of individuals to unique SW contours. Elsewhere, the distinction between SWs of individual dolphins following long-term acoustic study has allowed researchers to successfully recognise SWs within acoustic datasets of sufficient size [20].

Conversely, males seek social kinship away from their mothers, forming alliances for the purpose of future reproductive success [37]. This social distance prevents the need for males to distinguish their SWs from their female relatives’, resulting in mothers and their sons possessing similar SW contours. Further, male dolphins have been found to converge their SW structure with allied males [57]. Thus, identifying male SWs proves challenging. In this study, the positioning of male or female subgroups relative to the hydrophone was not considered. Some of the 22 SW contours may therefore represent assemblies of mother and son, or allied male, whistles. Indeed, far fewer SW contours than individual dolphins were identified, likely indicating the lumping of indistinguishable unique SW contours by researchers. In future studies, recording the SWs of male and female subgroups separately should be attempted.

### 4.3. Resident–Transient

Large standard deviations in SW contour modulations (numbers of extrema, inflection points, and steps) may reflect the broad range of selective acoustic pressures experienced by GL individuals of differing philopatric habits. Transient animals would be exposed to a more complex soundscape while transiting through the Bass Strait. Along their way, they likely encounter greater numbers of non-conspecific vocalising cetaceans than do their resident counterparts [58], influencing transient male SW composition. The evolution of SW complexity within the resident GL population may therefore be driven by periodic influxes of novel transient male SWs [59]. Indeed, transient GL animals indicated the greatest conditionally probable association with the widest variety of potential SW contours, seen in Clusters A and B, as well as individuals with a distinctly strong association with distinct SW contours (Section 3.4). Additionally, a few resident animals showed a strong association with diverse SW contours. Both resident pairs showed conditionally probable association with one contour type each (Section 3.4), reflecting reduced contour variability between resident individuals. The interaction between GL transients and other cetaceans migrating and transitioning through Bass Strait may force greater whistle variation in these animals, in an effort to retain their ability to be identified sonically by their conspecifics and differentiate themselves from sympatric non-conspecific vocalisers [60].

### 4.4. SW in PAM

Abundance estimates and spatial pattern mapping are being undertaken on other *Tursiops* spp. populations by employing the SIGID method to detect the presence and quantity of unique SW contours in PAM datasets [18,22]. Functional population size computed from photographic mark-recapture studies has been found to reflect that evaluated by quantifying the number of district SW contours recorded [61,62]. Interestingly, SWs are not always recorded at all locations within a PAM array [63], providing insight into the variation in habitat function at these sites. Both known residential populations of the Burrunan dolphin require ongoing monitoring, with consideration of functional population size and demographics included in a localised conservation management strategy. Coupling increased acoustic monitoring efforts with ongoing visual observation could provide increased insight into the behavioural ecology and survivability of this species.

## 5. Conclusions

This study provides the first analysis of SWs within the GL Burrunan dolphin population, laying the groundwork for individual- and subgroup-level identification using PAM. Despite challenges in directly attributing SW contours to individual dolphins, strong associations between distinct SW contours and highly associated social clusters and pairs offer valuable insight into population dynamics. Further study should consider the effectiveness of recording the vocalisations of solitary animals or spatially isolated mother–calf pairs for individual-level SW determination. The findings underscore the value of combining acoustic and visual data to monitor small, threatened cetacean populations. As PAM continues to expand into new regions, this integrative approach will be essential for improved understanding of dolphin ecology and long-term conservation planning.

## Figures and Tables

**Table 1:** Descriptive statistics, including mean, standard deviation (std), range (min–max), and percentiles (10–90%), of measured whistle parameters, including start, end, minimum, and maximum frequencies; duration; and numbers of extrema, inflection points, and steps.

	Start Frequency [kHz]	End Frequency [kHz]	Min Frequency [kHz]	Max Frequency [kHz]	Duration [s]	Extrema [#]	Inflection Points [#]	Steps [#]
mean	5.91	7.97	4.24	12.43	0.44	1.87	1.31	1.13
std	2.33	3.83	1.88	3.98	0.25	2.14	1.87	2.45
min	0.84	2.06	0.61	5.18	0.04	0	0	0
10%	3.59	4.13	1.97	6.71	0.10	0	0	0
25%	4.69	5.44	2.45	9.15	0.26	0	0	0
median	5.63	6.80	4.38	12.63	0.41	1	0	0
75%	6.94	9.70	5.65	15.70	0.63	4	3	0
90%	8.52	13.78	6.65	17.57	0.70	4	3	5
max	17.53	21.56	8.59	22.34	1.34	11	10	16

## Data Availability

The raw data supporting the conclusions of this article will be made available by the authors on request.

## Supplementary Materials

The following supporting information can be downloaded at: https://www.mdpi.com/article/10.3390/ani15223259/s1, Figure S1: Visual representation of decision workflow of integrated photo-ID/acoustic data collection; Figure S2: Histograms of the conditional probability of any of the identified dolphins being photographed when each SW contour was recorded; Table S1: Summary of data collection effort; Table S2: List of dolphins sighted and photographically identified with the number of sightings in which each was encountered, their sex (if know), and their residency pattern (if known); Table S3: List of SW contours identified, a written description, an example spectrogram, the number of sightings in which each was recorded and visually confirmed (no. sightings), and the frequency of occurrence within recordings (Freq. = number of measured whistles of a SW contour/total extracted whistles * 100).
