# Supplementary material for: Acoustic Identity: Linking Signature Whistles and Visual Identification in a Threatened Dolphin Population
Source: Animals (Basel). 2025 Nov 10;15(22):3259. doi: 10.3390/ani15223259 (PMC12649472; doi:10.3390/ani15223259)
Supplement: Supplementary file 1 [file animals-15-03259-s001.zip › animals-3922575-supplementary.pdf]

Supplementary Material

Acoustic identity: linking signature whistles and visual identification in a threatened dolphin population

Amber Crittenden, Kate Robb, Christine Erbe

2. Methods

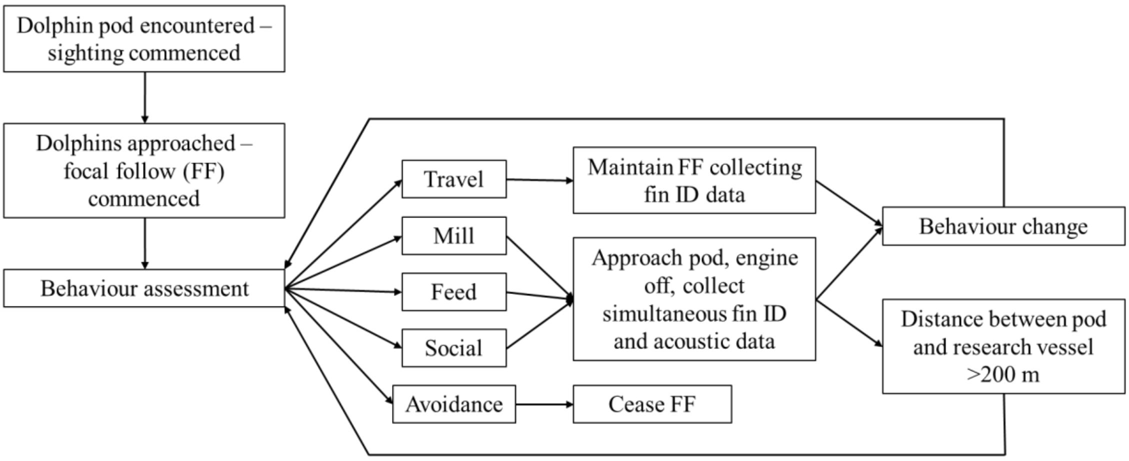

Figure S1: Visual representation of decision workflow of integrated photo-ID/acoustic data collection.

3. Results

Table S1: Summary of data collection effort.

Sighting	Date	Sighting duration [h:mm:ss]	No. acoustic files	Total record length [h:mm:ss]	No. SW contours	No. raw images	No. dolphins
1	14/02/2021	1:01:00	1	0:04:11	2	130	8
2	15/09/2021	2:18:00	1	0:04:36	1	799	15
3	2/10/2022	1:26:00	2	0:12:03	5	634	10
4	7/10/2022	2:55:00	2	0:16:43	4	242	7
5	7/10/2022	2:06:00	3	0:33:56	1	462	12
6	15/05/2023	1:35:00	2	0:14:41	2	107	15
7	23/07/2023	1:27:00	1	0:14:13	3	562	30
8	25/07/2023	1:47:00	1	0:01:52	1	118	5
9	8/10/2023	2:51:00	2	0:07:11	5	566	20
10	9/10/2023	1:37:00	1	0:03:28	2	500	18
11	10/10/2023	2:08:00	1	0:02:28	1	733	9
12	1/06/2024	2:05:00	1	0:09:46	2	176	14
13	25/07/2024	2:13:00	4	0:15:19	10	1,139	19

Table S2: List of dolphins sighted and photographically identified with the number of sightings in which each was encountered, their sex (if know), and their residency pattern (if known).

Dolphin ID code	No. sightings	Male (M) Female (F) Unknown (U)	Resident (R) Transient (T) Unknown (U)
GL103	1	F	T
GL105	2	M	R
GL107	10	M	R
GL111	10	F	R
GL116	2	F	R
GL117	4	M	R
GL204	2	U	T
GL205	1	F	R
GL207	1	F	T
GL208	2	U	U
GL209	1	M	R

GL210	2	M	R
GL211	1	F	T
GL212	8	F	R
GL216	2	U	T
GL218	4	F	R
GL220	1	U	U
GL302	1	F	T
GL308	5	F	R
GL311	3	M	R
GL312	4	M	R
GL313	1	U	T
GL314	1	U	U
GL315	1	U	R
GL10111	3	M	T
GL10116	2	U	R
GL10124	2	U	R
GL10202	5	F	R
GL10203	6	F	R
GL10206	3	M	R
GL10207	1	F	T
GL10208	2	F	T
GL10306	1	F	R
GL10307	6	F	R
GL10311	4	F	R
GL10314	1	F	T
GL10315	6	F	R
GL10316	1	F	R
GL10318	1	U	T
GL10402	3	F	R
GL10407	3	M	R
GL10408	3	F	R
GL10409	3	F	R
GL10414	9	M	R
GL10415	8	M	R
GL10417	10	M	R
GL10418	7	F	R
GL10419	4	F	T
GL10421	2	M	T
GL10423	3	M	T
GL10432	1	F	R
GL10436	2	M	T
GL10443	1	U	T
GL10445	2	F	T
GL10448	5	M	R
GL10450	1	U	T
GC01	1	M	R

*Table S3: List of SW contours identified, a written description, an example spectrogram, the number of sightings in which each was recorded and visually confirmed (no. sightings), and the frequency of occurrence within recordings (Freq. = number of measured whistles of a SW contour / total extracted whistles * 100).*

SW contour	Description	Spectrogram	No. sightings	Freq.

BConvex	Broadband convex of short duration	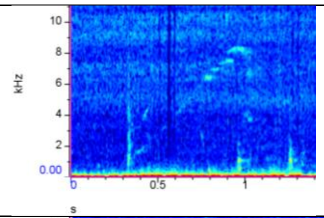	1	1.27
BuzzConvex	Convex with biphonic buzz <2 kHz during local maximum	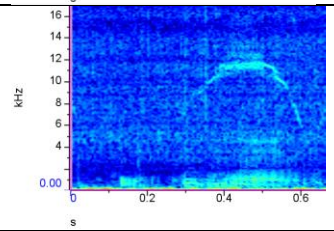	1	5.08
BuzzUp	Narrowband buzz followed immediately by an upsweep	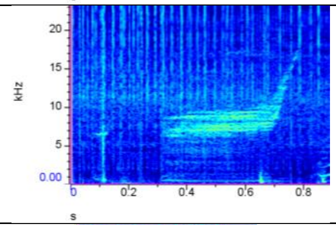	1	2.54
ConstUp	Almost flat (constant), narrowband upsweep	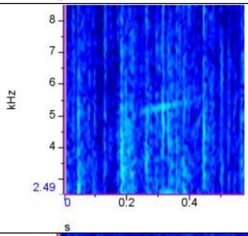	1	1.69
FlatConvex	Convex whistle with local maximum of extended constant frequency	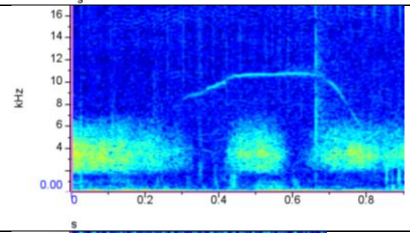	3	3.81
HookSine	A sine whistle with two extrema, the second of which is a local maximum of short duration	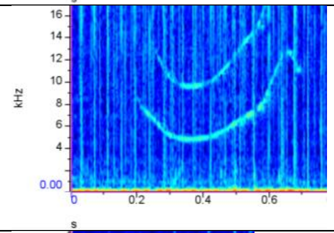	1	1.69
HookUp	Local minimum followed by upsweep	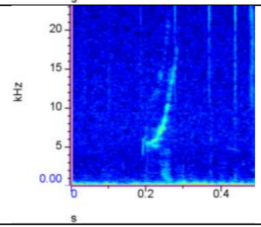	3	5.08
InflConvex	Stepped convex with 2 inflection points, 1 before and 1 after the extremum (local maximum)	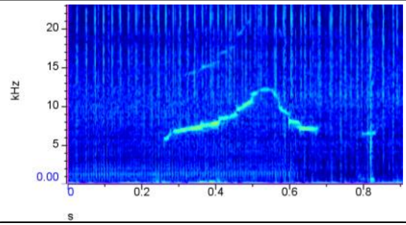	1	1.27

InflUp	Upsweep with at least 2 inflection points	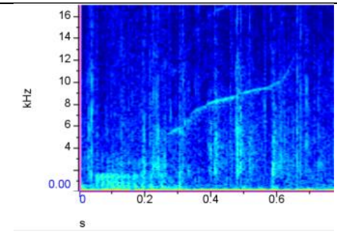	2	6.36
LongUp	A broadband, short-duration upsweep, may or may not have steps	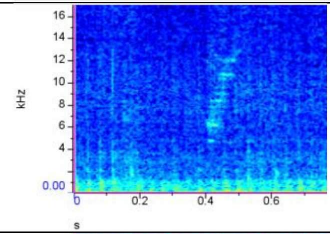	4	7.20
LowUp	Lower-frequency, short-duration upsweep	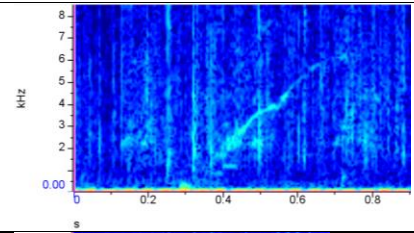	2	5.08
MBuzz	Sine whistle with 4 extrema, with a biphonic buzz in final extremum (local minimum)	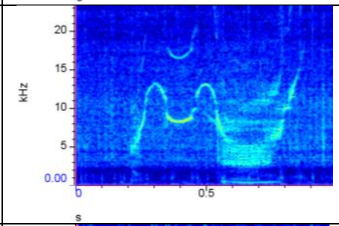	4	26.27
MidUp	Mid-frequency short-duration upsweep, may or may not have steps	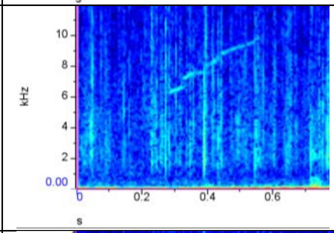	1	1.27
MultiL	Multi-looped sine with 5 or more extrema	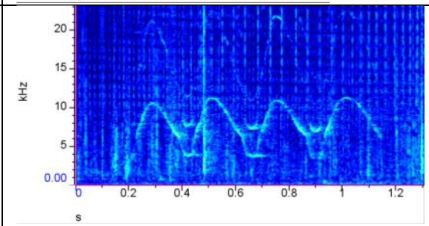	1	4.24
NUp	Very narrowband, short-duration upsweep	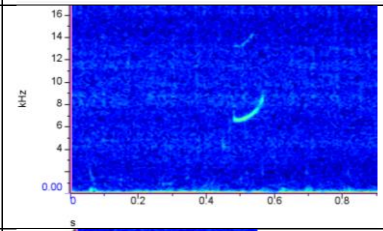	2	4.24
ShortConvex	Narrowband convex	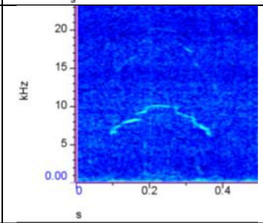	2	5.08

SmoothConvex	Non-stepped broadband convex	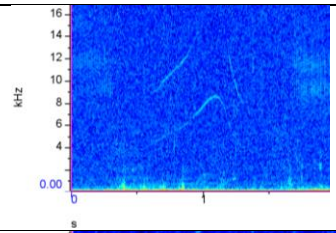	1	1.27
StepConvex	Broadband convex with 5 or more steps	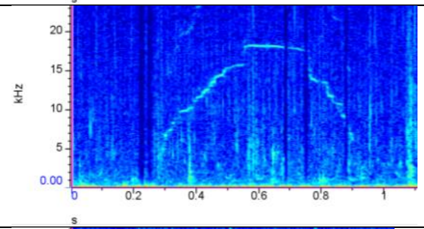	2	2.54
StepSine	Sine with 3 or more extrema with steps and no biphonics	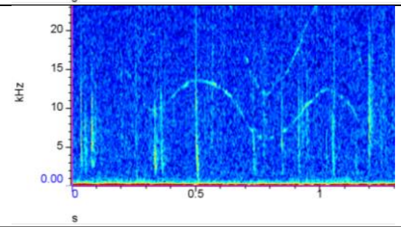	1	0.85
UpFlat	Upsweep with final section of reduced gradient	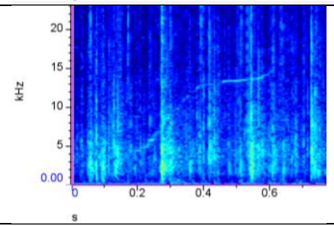	1	0.85
WUp	Upsweep with a local minimum, 2 inflection points, and 3 extrema, forming a flattened diagonal "W" shape	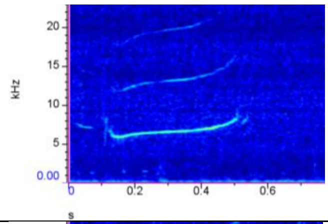	2	3.39
Zup	Smooth upsweep with an inflection point to form a horizontal "Z" shape	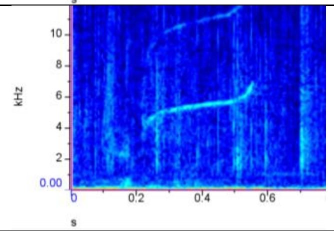	2	8.90

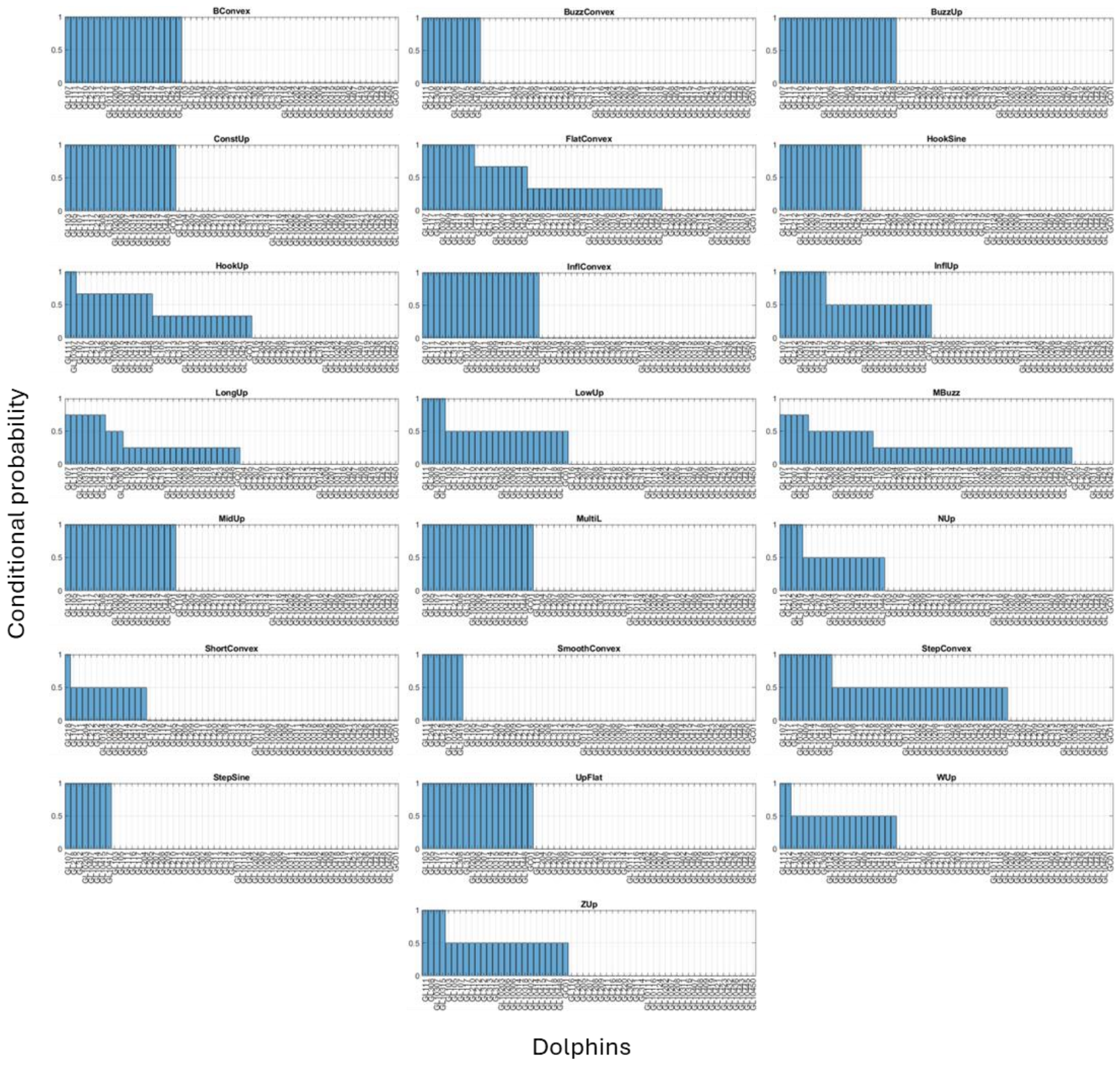

Figure S2: Histograms of the conditional probability of any of the identified dolphins being photographed when each SW contour was recorded.
